# Mortality among the homeless: Causes and meteorological relationships

**DOI:** 10.1371/journal.pone.0189938

**Published:** 2017-12-21

**Authors:** Jerzy Romaszko, Iwona Cymes, Ewa Dragańska, Robert Kuchta, Katarzyna Glińska-Lewczuk

**Affiliations:** 1 Family Medicine Unit, University of Warmia and Mazury in Olsztyn, Olsztyn, Poland; 2 Department of Water Resources, Climatology and Environmental Management, University of Warmia and Mazury in Olsztyn, Olsztyn, Poland; 3 Municipal Social Welfare Center in Olsztyn, Olsztyn, Poland; Columbia University, UNITED STATES

## Abstract

**Background:**

The homeless constitute a subpopulation particularly exposed to atmospheric conditions, which, in the temperate climate zone, can result in both cold and heat stress leading to the increased mortality hazard. Environmental conditions have become a significant independent risk factor for mortality from specific causes, including circulatory or respiratory diseases. It is known that this group is particularly prone to some addictions, has a shorter life span, its members often die of different causes than those of the general population and may be especially vulnerable to the influence of weather conditions.

**Materials and methods:**

The retrospective analysis is based on data concerning 615 homeless people, out of which 176 died in the analyzed period (2010–2016). Data for the study was collected in the city of Olsztyn, located in north-east Poland, temperate climatic zone of transitional type. To characterize weather conditions, meteorological data including daily minimum and maximum temperatures and the Universal Thermal Climate Index (UTCI) were used.

**Results:**

The average life span of a homeless person was shorter by about 17.5 years than that recorded for the general population. The average age at death of a homeless male was 56.27 years old (SD 10.38), and 52.00 years old (SD 9.85) of a homeless female. The most frequent causes of death were circulatory system diseases (33.80%). A large number of deaths were attributable to smoking (47.18%), whereas a small number was caused by infectious diseases, while a relatively large proportion of deaths were due to tuberculosis (2.15%). Most deaths occurred in the conditions of cold stress (of different intensity). Deaths caused by hypothermia were thirteen-fold more frequently recorded among the homeless than for the general population. A relative risk of death for a homeless person even in moderate cold stress conditions is higher (RR = 1.84) than in thermoneutral conditions.

**Conclusions:**

Our results indicate excessive mortality among the homeless as well as the weak and rather typical influence of atmospheric conditions on mortality rates in this subpopulation, except for a greater risk of cold related deaths than in the general population. UTCI may serve as a useful tool to predict death risk in this group of people.

## Introduction

The average life span in the general population keeps increasing in Europe. In Poland in 2015 it was on average 73.6 years for males and 81.6 years for females [1]. These values could possibly be higher if not for excessive mortality in specific social groups. According to available literature, the subpopulation of homeless people constitutes such a social group. Nusselder WJ et al., based on data collected in Rotterdam (the Netherlands), claim that homeless men and women in Rotterdam had a 3.5 times higher mortality rate as compared to their peers in the city′s general population and the disparity in mortality between homeless and non-homeless people was greater for women. The life expectancy of a homeless person is several years shorter depending on age and sex of an analysed homeless group [2]. Similarly Vuillermoz C et al., when analyzing excessive mortality of the homeless in France, based on data from 2008–2010, observe that the average life span of a homeless person amounts to only 49 years, thus being 28 years shorter than in the general population [3]. Data obtained in North America are quite similar. Hwang SW et al. noted that the average life span of a homeless person in Toronto was 48 years, whereas in the Baggett TP et al. study conducted in Boston it was 51 years [4, 5]. These findings are intriguing because they suggest the universality of this phenomenon. Places where the aforementioned studies were conducted are located in different countries, characterized by different models of social care and different climate, but they share the quite similar demographic characteristics of the homeless subpopulation. Homeless people do not constitute some separate category, but are derivatives of societies in which they live. Although American and West European authors indicate evident over-representation of racial and ethnic minorities among the homeless, this is not observed in Central European countries, slightly less attractive as the target for emigration [6]. Moreover, the homeless described by American and West European authors are generally slightly younger than those reported by researchers from Central and Eastern Europe [7, 8]. This seems to be related to the etiology of homelessness. Although various addictions are not the only cause of homelessness, yet addictive behaviors do contribute to it [9]. McVicar D et al. generalize that “We find that the two are closely related: homeless individuals are more likely to be substance users and substance users are more likely to be homeless” [10]. However, the type of the active substance used depends on its local availability and, more importantly, on the age of homeless people. Younger homeless people are more often reported to be drug users, whereas alcohol addiction dominates in older age groups [11]. As for both mentioned reasons (availability and preferences), drug addiction is more frequent in American findings, whereas alcoholism dominates in Europe [12]. This correlation is also noticeable in Poland, where the homeless subpopulation is evidently older and definitely more often dependent on alcohol than drugs. In a study conducted in Olsztyn, Poland, Romaszko et al. estimated the percentage of the homeless addicted to alcohol at more than 78%, and the average age of the studied group at 55 years of age [8]. These values suggest that statistically rates of early mortality should be rather smaller than larger as compared to data obtained in the West. Pathology that appears in older age groups should have a statistically weaker impact on the average age at death.

Many studies emphasize a correlation between mortality in the general population and meteorological parameters. In a meta-analysis of data encompassing 12 countries, Bunker A et al. indicate that both the increase and decrease of temperature by 1°C may result in the elevated mortality rates of elderly patients owing to cardiovascular reasons [13]. In a large study of 6 513 330 deaths in 50 American cities, Medina-Ramón M et al. reveal a statistically significant correlation between mortality and extreme cold and extreme heat factors [14]. Based on data for the period 2006–2011 (China), Huang Z et al. arrive at a similar conclusion [15]. It appears that changeable temperatures in the surrounding environment may have an impact on mortality rates in the general population irrespective of the place of study. It may be hypothesized that in countries located in a cold climate, cold is the major risk factor for elevated mortality rates, whereas in subtropical countries it is heat. This hypothesis is, however, incorrect. When analyzing 74 225 200 deaths over a period of 27 years in 384 locations, Gasparrini A et al. observed that cold is a stronger risk factor for death [16]. In a similar analysis involving data from subtropical locations (6 214 deaths), Dang TN et al. note that although high temperature is a risk factor for death in short lags, it is cold that is a much stronger predictor of deaths in long lags [17]. Although the relationship between various health problems and mortality during unfavorable meteorological conditions is quite well-known and reported, the apparently obvious correlation with socio-economic conditions raises more doubts. In northern Europe the first wave of cold weather is normally associated with articles in the daily press devoted to the difficult situation of the homeless. Yet, data based on scientific findings are not so unequivocal. Although Hales S. et al. note higher mortality rates in wintertime among a low income population in New Zealand, neither Wilkinson P et al. (data from the UK) nor Rau R. (data from Denmark) confirm such a correlation [18–20]. Narrowing this literature overview down to the homeless significantly limits the number of available publications. Based on data from Paris, Rouquette A et al. revealed that 61.7% of people cared for at emergency departments in wintertime were homeless [21]. According to a study conducted by Ohsaka T in Osaka (Japan), hypothermia was the cause of death among the homeless in 4.08% cases [22]. Based on a study conducted in Ontario (Canada), Chen H. et al. suggest that the homeless are particularly vulnerable to meteorological conditions, but on the basis of the obtained data this suggestion cannot be confirmed [23]. Taking all the aforementioned studies and findings into consideration, we decided to verify whether the homeless are especially at risk of increased mortality and whether their mortality levels change depending on local meteorological conditions.

## Materials and methods

### Study population

The retrospective analysis was based on data collected by the Shelter for the Homeless in Olsztyn (Poland) recording people who stayed overnight in the shelter in the period 2010–2016. Olsztyn is inhabited by about 175 000 residents, including approximately 150–200 homeless people. The number of homeless individuals is generally stable, determined annually by the point in time method and monitored by the Regional Centre for Social Policy (The Marshall Office). The database of people who stayed overnight (at least once) in the shelter during the studied period included records for 1463 individuals. From this number, we managed to follow up on 615 cases (individuals who could be identified on the basis of the Personal Identification Number PESEL–absolute identifier—the national identification number used in Poland, mandatory for all permanent residents of Poland and for temporary residents living in Poland for over 2 months) and noted 176 deaths in the analyzed period. The causes of death of the homeless were identified based on information provided by the Central Statistical Office for the period 2010–2015 and encompassing the data of 146 persons (data for 2016 will be available in the following year).

### Meteorological data

To establish weather conditions in Olsztyn in the analyzed period, meteorological parameters recorded by the Institute of Meteorology and Water Management in Poland were examined. To identify non-neutral thermal conditions for people, characterized by very high or very low air temperatures, data embracing daily maximum and minimum air temperatures were employed and the numbers of hot (tmax ≥ 25.0°C), very hot (tmax ≥ 30.0°C), cold (tmax < 0.0°C), very cold (tmin ≤ -10.0°C) and extremely cold (tmax ≤ -10.0°C) days were determined.

Data collected regularly at 6.00, 12.00, and 18.00 UTC (Coordinated Universal Time) recording air temperature, relative humidity of air, actual vapour pressure, cloudiness and wind speed were applied to calculate the Universal Thermal Climate Index (UTCI). UTCI is derived from the analysis of human thermal balance, conducted with the use of a multi-node model of thermoregulation that calculates human heat transfers within the body and between the human body and the surrounding air [24, 25]. UTCI provides information on factual body thermoregulation processes that are dependent on external meteorological conditions. The values of UTCI are classified considering the intensity of organism physiological reactions in specific meteorological conditions and this is associated with the necessity to use various protective measures against organism overheating and hypothermia. Heat stress occurs on days featuring high air temperature and intensive sunlight, whereas cold stress dominates on days characterized by low air temperature and high wind speed. Out of the UTCI values determined for 6.00, 12.00 and 18.00 UTC, the value indicating the most adverse conditions was adopted to assess the body thermal stress on a particular day. Conditions sufficient to retain thermal comfort were defined as such situations when at each measuring time UTCI values indicated the lack of thermal stress, thus being in the range of 9.1°C and 26.0°C.

### Statistical analysis

The data were statistically analyzed to find out the relationship between deaths among the homeless and meteorological variables. The comparison of deaths recorded for this population in seasons was performed using the non-parametric Kruskall-Wallis test at p < 0.05. The explanatory variables (tmax, tmin, UTCI) were employed for the Generalized Liner Model (GLM) construction assuming a Poisson distribution and using the logarithm as the link function [26]. The contribution of the predictors was evaluated by testing individual coefficients with Wald’s test at p<0.05. Additionally, we examined relationships between biothermal indices and deaths in two groups of diseases: group A included deaths related to typical pathologies for the homeless subpopulation such as: smoking-attributable, alcohol and cold, external cause, alcohol only, violence and cold only; group B embraced deaths classified by the European Shortlist for Causes of Death including diseases of the circulatory system, external causes of injury and poisoning, diseases of the respiratory system, neoplasms and diseases of the digestive system.

Although most studies have quantified the association of mortality with ambient temperature in terms of relative risk (RR), only a few studies have assessed the homeless mortality burden attributed to cold/hot temperatures while the use of UTC index seems underestimated in cohort studies [16, 27, 28].

Based on UTCI values, we calculated the relative risk to quantify the magnitude of the cold and heat stress as factors influencing mortality among the homeless (outcome). The relative risk understood as a ratio of risk outcome with factor present (cold or heat stress) to risk outcome with factor absent (thermoneutral conditions, no thermal stress considered as a control group) was considered [29]. A RR number between 0 and 1 implies that risk of mortality among the homeless is lower, while RR >1 implies that the mortality risk is grater when the cold or heat stress is present. For the relative risk observed to be considered statistically significant, the 95% confidence interval (CI) was calculated.

To assess homeless deaths caused by the diseases assigned to groups A and B in relation to monthly UTC Index we performed a Two-way Cluster Analysis (TWCA) [30]. Cluster analysis is a statistical technique designed to classify samples into groups based on the degree of similarity among them with respect to a defined set of variables. The clusters were computed with the use of a Euclidean distance measure and Ward’s linkage method. TWCA was applied to the data set using PC-ORD 6.0 software (PC-Ord 6.0, Gleneden Beach, Oregon). Power transformation of data was necessary to avoid negative values of meteorological variables. Due to standardization of the data, each parameter contributed equally to the data set variance and carried equal weight in the analysis.

Statistical analyses were performed with STATISTICA 13.1 for Windows.

### Ethic statement

The study protocol was approved by the Bioethical Committee of the Warmia and Mazuria Regional Medical Chamber in Olsztyn (239/17/Bioet). The authors declare no conflicts of interest in relation to this article.

## Results

### Demographic section

In the analyzed group of 176 deceased homeless people, 163 (92.61%) were men. The basic demographic data, including gender, age at death and date of death, are presented in Table 1.

**Table 1:** Demographic data of the deceased homeless.

	n	%	mean age	SD
total number of deaths	176	100	55.95	10.37
females	13	7.38	52.00	9.85
males	163	92.61	56.27	10.38
number of deaths per years				
2010	35	19.89	54.14	9.50
2011	26	14.77	54.31	12.12
2012	16	9.09	51.56	10.73
2013	28	15.91	58.18	10.11
2014	21	11.93	55.57	9.11
2015	22	12.50	59.86	11.27
2016	28	15.91	57.25	9.12
number of deaths per month (n = 174)*				
January	24	13.79	55.21	10.04
February	13	7.47	55.77	9.72
March	14	8.05	49.79	11.98
April	13	7.47	61.46	12.45
May	8	4.60	59.88	8.43
June	7	4.02	58.14	4.63
July	16	9.20	56.63	8.29
August	14	8.05	58.21	9.86
September	11	6.32	61.09	8.08
October	16	9.20	51.94	9.67
November	17	9.77	58.71	11.76
December	21	12.07	51.14	10.14

* In two cases it was impossible to establish the date of death.

According to data obtained from the Central Statistical Office for the city of Olsztyn in the period 2010–2015, the average age at death for an adult person (over 18 years of age) was 73.45 years old, SD 14.77 (n = 8647); 69.83 years old, SD 14.91 (n = 4468), for men and 77.33 years old, SD 13.59 (n = 4179), for women, respectively. These values are higher (in each comparison p<0.001) than the ones observed in our study (Table 1). According to the same data (the Central Statistical Office) the average number of deaths per 1000 inhabitants is 9.86 annually. In our study we followed up on 615 people for 7 years and noted 176 deaths. This amounts to the level of mortality of 40.88 (calculated per 1000 per year), thus being four-fold higher than in the general population. The average age at death is 56.27 years old for a homeless male and 52.00 years old for a homeless female; the difference is not statistically significant (p = 0.08).

Out of 148 cases of death (period of 2010–2015), in which it was possible to obtain information on the cause of death, it proved impossible to determine the cause of death in 6 cases. The remaining 140 cases were analyzed quantitatively, identifying groups of causes according to the European Shortlist for Causes of Death [31], and dividing them into subgroups of interest to our study, such as: alcohol related deaths [32], smoking-attributable deaths [33], cold related deaths–consistent with ICD–10 codes X31 or T68 (exposure to excessive natural cold, hypothermia), deaths due to external causes and violent deaths [34]. These results are presented in Table 2.

**Table 2:** Causes of death (n≥5).

	n*	%	mean age	SD
Total death account	142	100.00	55.61	10.57
Group A diseases—related to typical pathologies for homeless subpopulation				
Smoking-attributable deaths	67	47.18	57.48	10.20
Alcohol related death + cold	33	23.24	51.12	10.11
External Cause	30	21.13	50.17	9.73
Alcohol related death	28	19.72	50.36	10.73
Violent Death	18	12.68	49.00	11.06
Cold related death	5	3.52	55.40	3.78
Group B diseases—European Shortlist for Causes of Death				
Diseases of the circulatory system	48	33.80	57.38	10.26
External causes of injury and poisoning	30	21.13	50.17	9.73
Diseases of the respiratory system	16	11.27	58.19	13.34
Neoplasms	16	11.27	62.50	5.02
Diseases of the digestive system	15	10.56	53.67	7.37

*Multiple diagnoses are possible in group A diseases, for instance, alcohol related death is a subset of alcohol related death + cold.

What draws attention here is the relatively small number (n = 4) of deaths due to infectious diseases and at the same time the relatively large number (n = 3) of deaths due to tuberculosis.

### Weather conditions

**Fig 1:**
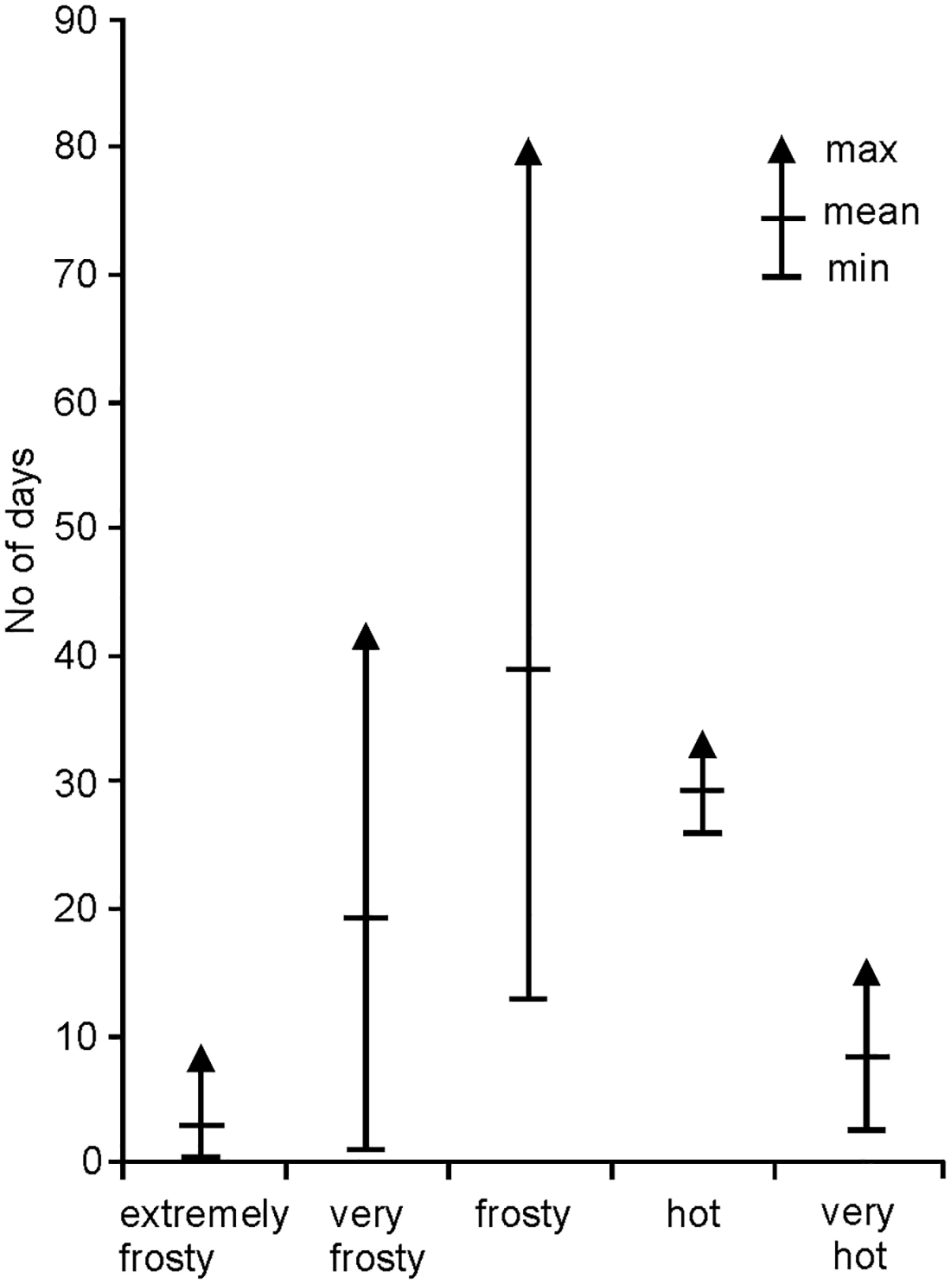
Number of characteristic days in 2010–2016 by thermal categories.

In the analyzed period hot and very hot days occurred from April till September, with the largest number in July, their mean number amounting to 29 and 9 days, respectively. Frosty, very frosty and extremely frosty days, characteristic for the cold season, were most often noted in December, January and February. Their average number per year was 39, 20 and 4 days, respectively (Fig 1).

Biothermal conditions with no thermal stress amounted on average to 28% days per year. The largest numbers of such days (more than 50% per month) were observed from April till September, with the maximum in June when their number reached almost 73% (Table 3)

**Table 3:** Frequency (%) of thermal stress categories determined on the basis of UTCI.

Month / UTCI[°C]	cold stress					thermoneutral zone	heat stress			
	extreme <−40.0	very strong -39.9 to -27.0	strong -26.9 to -13.0	moderate -12.9 to 0.0	slight 0.1 to 9.0	9.1 to 26.0	moderate 26.1 to 32.0	strong 32.1 to 38.0	very strong 38.1 to 46.0	extreme >46.0
January	-	1.4	35.9	61.8	0.9	-	-	-	-	-
February	-	0.0	24.2	69.2	6.6	-	-	-	-	-
March	-	0.5	6.9	61.8	29.5	1.4	-	-	-	-
April	-	-	1.4	26.7	49.0	22.9	-	-	-	-
May	-	-	-	8.3	36.4	54.4	0.9	-	-	-
June	-	-	-	-	1.4	72.4	21.4	4.8	-	-
July	-	-	-	-	0.9	53.5	32.3	12.9	1.0	-
August	-	-	-	-	-	61.3	28.1	9.7	2.0	-
September	-	-	-	3.3	31.4	65.2	-	-	-	-
October	-	-	0.5	25.8	64.1	9.7	-	-	-	-
November	-	-	3.3	64.8	31.4	0.5	-	-	-	-
December	-	0.9	22.6	73.3	3.2	-	-	-	-	-

Almost 63% of days per year were classified as characterized by cold stress, whereas the most frequently noted category was moderate cold stress, on average occurring on 33% of days per year. Days classified as those of moderate and strong heat stress occurred from May till August and on average they amounted to 9% of days per year in total. No cases of unbearable cold/heat stress were noted.

**Fig 2:**
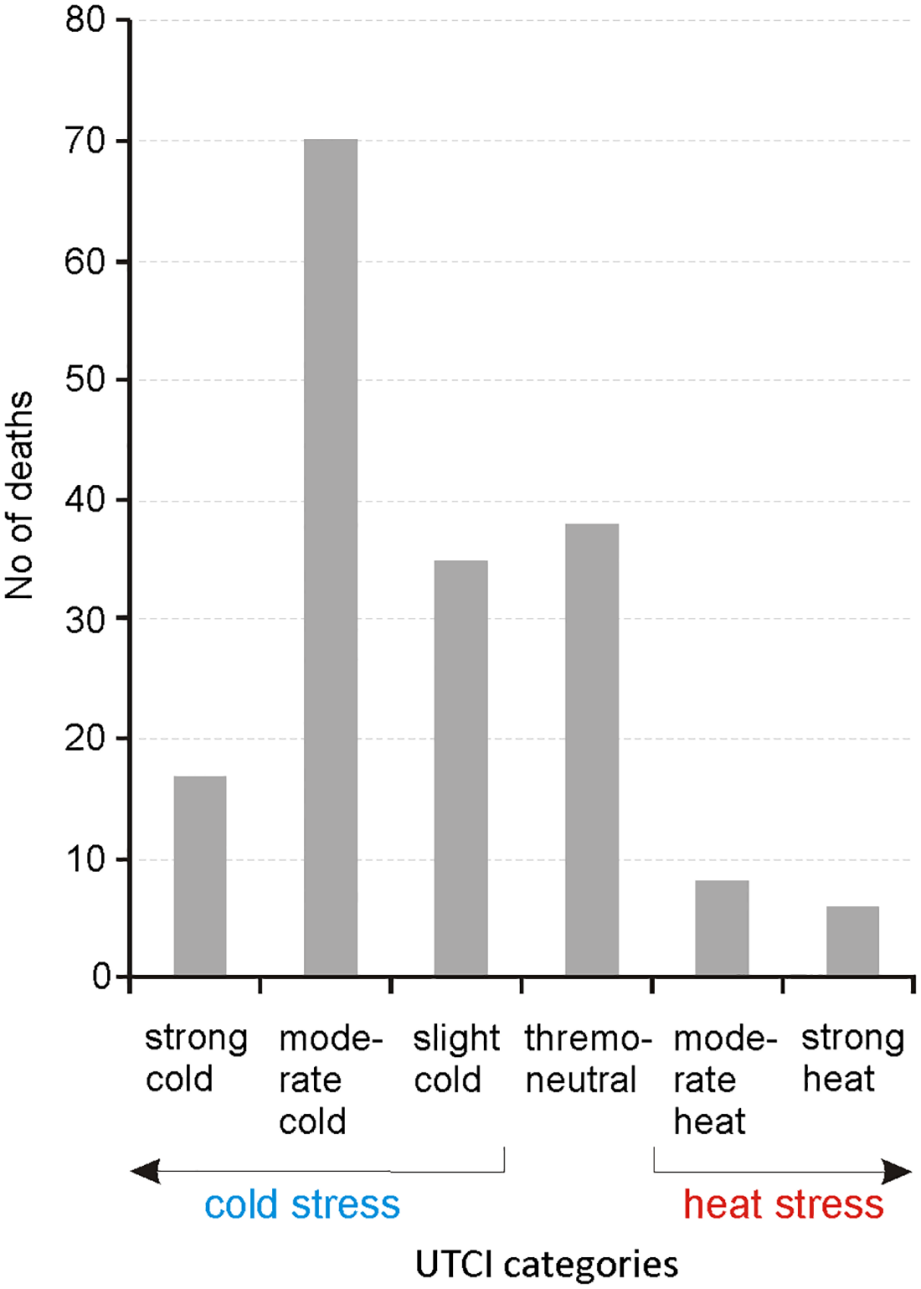
Number of deaths in categories of thermal stress according to UTCI (n = 174).

The largest number of deaths, approximately 40% (n = 70), occurred during moderate cold stress, i.e., the most frequently observed thermal stress, whereas 22% (n = 38) happened in thermoneutral conditions; 9% (n = 17) of deaths occurred in strong cold stress conditions, whereas 8% (n = 14) in heat stress conditions (Fig 2).

Analysis of variance performed for mortality among the homeless in the categories of UTCI stress created groups of means not significantly different in the Kruskal-Wallis test (p = 0.108 > p = 0.05; not shown in the graph).

Mortality rates among the homeless under the cold stress at UTCI ≤ 8.8°C (Table 4) show the highest relative risk in comparison to deaths among the homeless occurring during thermoneutral conditions, where UTCI ranged from 9.2°C to 25.8°C (RR = 3.21 within 95%CI 2.38–4.32; p < 0.001). This indicates a significant risk of excessive mortality among the homeless under the cold stress relative to mortality among the homeless under the thermal comfort.

**Table 4:** Relative risks (RR) of mortality among the homeless population due to cold and heat stress (exposed group) in comparison to thermoneutral conditions (control group). The RR and 95% confidence interval (CI) are calculated according to Altman 1991 [29].

Homeless mortality cause	Relative risk	95% CI	P-value
**Cold stress vs thermoneutral conditions**	3.21	2.38 to 4.32	p < 0.001*
Strong cold stress vs thermoneutral conditions	0.45	0.26 to 0.76	**p = 0.003**
Moderate cold stress vs thermoneutral conditions	1.84	1.32 to 2.57	**p < 0.001**
Slight cold stress vs thermoneutral conditions	0.92	0.61 to 1.38	p = 0.693
**Heat stress vs thermoneutral conditions**	0.37	0.21 to 0.66	**p < 0.001**
Moderate heat stress vs thermoneutral conditions	0.22	0.10 to 0.45	**p < 0.001**
Strong heat stress vs thermoneutral conditions	0.17	0.07 to 0.38	**p < 0.001**

*Significant p-values are marked in bold

Among three groups of mortality among the homeless under the cold stress, the highest probability of death was revealed for moderate cold stress (RR = 1.84; **p < 0.001**), which markedly differed from the probabilities for the strong cold stress and slight cold stress: 0.45 (p = 0.003) and 0.92 (p = 0.693, not significant), respectively.

Compared with mortality rate of the homeless exposed to the cold stress, the heat stress (UTCI ≥ 26.7°C) posed a significantly lower RR for mortality among the homeless in relation to comfort weather conditions (RR = 0.37 within 95%CI 0.21–0.66; p < 0.001). The disparity in RR mortality between the homeless under heat stress and neutral thermal conditions was significantly higher (at p < 0.001) in the case of moderate (RR = 0.22) rather than strong heat stress (RR = 0.17).

Evidence from our study also suggests that both minimum and maximum temperatures (tmin, tmax respectively) might be meteorological risk factors for the homeless (Table 5). The GLM analysis indicates that these meteorological factors contributed to mortality among the homeless caused by alcohol consumption during cold periods (p = 0.005), the cold itself (UTCI, p = 0.041) as well as by external diseases (p = 0.004). Minimum temperatures were a statistically significant factor (p<0.001) in the case of respiratory system diseases. The role of seasons as a factor contributing to deaths among the homeless was deemed not statistically significant (both for group A and group B of diseases).

**Table 5:** Assessment of meteorological parameters used in the Generalized Linear Model (GLM) assuming the Poisson distribution with log link.

Parameter	n/k	tmin			tmax		UTCI		
		p	W		p	W	p		W
Total death account	1.0	**<0.001**	**11.90**		**0.011**	**6.50**	n.s.		-
**Group A diseases—related to typical pathologies for homeless subpopulation**									
Smoking-attributable deaths	2.23	**0.034**	**4.51**	**0.038**		**4.32**		n.s.	-
Alcohol related death + cold	4.45	**0.005**	**7.96**	n.s.		-		n.s.	-
External Cause	4.90	**0.004**	**8.30**	n.s.		-		n.s.	-
Alcohol related death	5.25	n.s.	-	n.s.		-		n.s.	-
Violent Death	8.17	n.s.	-	n.s.		-		n.s.	-
Cold related death	29.40	n.s.	-	n.s.		-		**0.041**	**4.19**
**Group B diseases—European Shortlist for Causes of Death**									
Diseases of the circulatory system	3.13	n.s.	-	n.s.		-		n.s.	-
External causes of injury and poisoning	4.9	**0.004**	**8.30**	n.s.		-		n.s.	-
Diseases of the respiratory system	9.19	**<0.001**	**11.76**	n.s.		-		n.s.	-
Neoplasms	9.19	n.s.	-	n.s.		-		n.s.	-
Diseases of the digestive system	9.80	n.s.	-	n.s.		-		n.s.	-

Comments: significant p-values are marked in bold; n–total number of deaths; k–number of deaths caused by individual disease; n.s.- no statistical significance (p>0.05)

It should be noted, however, that a small number of deaths due to hypothermia (n = 5) limits the value of these calculations. Cases of deaths caused by hypothermia occurred during the cold season; lowest temperatures ranged then from -17.0°C to +3.6°C, with UTCI values (from –12.2°C to –20.8°C) indicating thermal stress within the categories of moderate cold stress and strong cold stress, when actions to protect the body against hypothermia should be undertaken.

**Fig 3:**
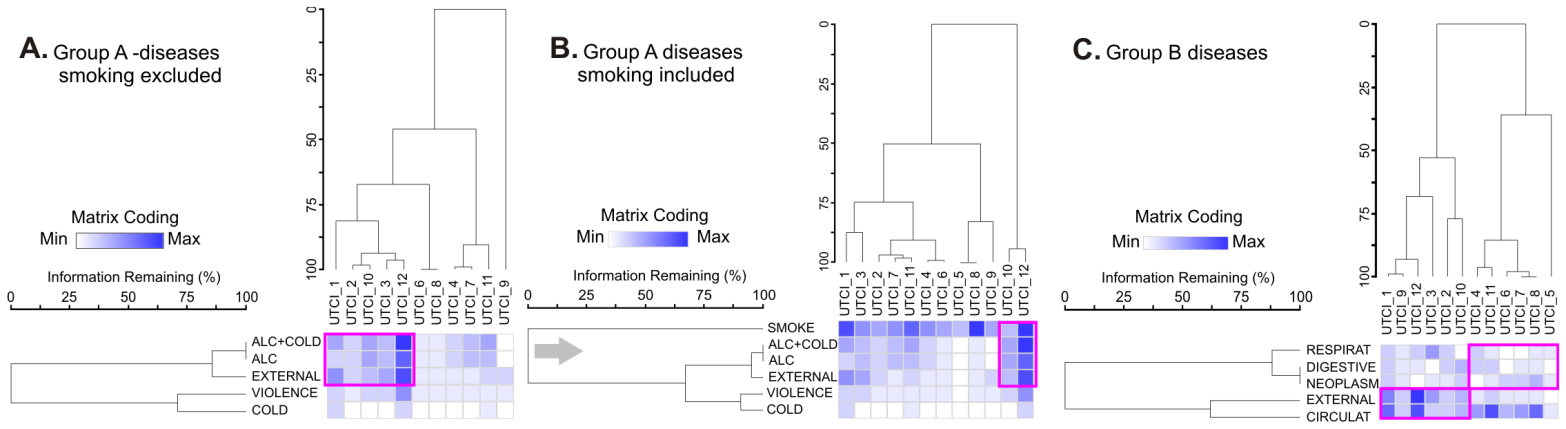
Two-way cluster (bi-cluster) diagram of the monthly UTC indices and a number of deaths among the homeless as a result of group A causes excluding smoking (A), group A causes with smoking (B) and group B causes (C). Abbreviations referring to causes of death correspond to those in Table 2. UTCI and cause clusters are based on Euclidean distance measures and Ward’s linking method. The shading of each box represents a relative contribution of the number of deaths in a column. Diseases abbreviations: see Table 2.

The analysis of mortality cases among the homeless indicated an association between monthly changes in the UTCI values and deaths due to group A and group B diseases. TWCA performed for group A diseases related to pathologies (Fig 3A) revealed that, apart from smoking, alcohol consumption combined with cold stress and external diseases created a cluster corresponding to the UTCI values observed in December and October. Smoking (Fig 3B) created an individual cluster that dominated over the analyzed causes of mortality among the homeless irrespective of a month. This finding supplements the results obtained from the GLM analysis (Table 5). In group B diseases (Fig 3C), circulatory problems and external causes of death created a single cluster that corresponded to the UTCI cluster consisting of autumn and winter months (January, February, March, September, October and December). This cluster differed from other diseases (respiratory, digestive and neoplasms) which were less frequent, particularly in spring and summer.

## Discussion

Literature sources referred to in the introductory section indicate that the average life span of a homeless person is shorter by 16–28 years than the values observed in the general population, and ranges from 48 to 51 years. In our study (Table 1) the average age at death of a homeless person was 55.95 years old and this value was smaller by 17.5 years than the one found in the general population. This difference is particularly evident in the group of females and amounts to 25 years (52.00 and 77.33, respectively), whereas the average age at death of a homeless female seems to be lower than in the case of males, inversely than in the general population. Considerable, although typical of this subpopulation, predominance of men makes this difference statistically insignificant p = 0.08 [35].

Excessive mortality among homeless females has been noted by other researchers, yet it has not been confirmed by all of them [36–41]. It seems that this problem refers to a younger age group in particular and, consequently, it is evident in the younger average age at death. Our empirical material does not warrant the analysis of the causes for this phenomenon. We believe, however, that further studies accounting for different courses and manners of becoming addicted as regards homeless females and males might explain this issue [42–44].

As specified by various reports, deaths among the homeless are primarily attributed to circulatory system diseases (23%), followed by drug overdose (21%) and accidents (14%) excluding poisoning [45]. Among young homeless people the most frequent causes of death include poisoning (opiates, alcohol), as well as accidents and suicides, whereas in the slightly older age group (25–44 years), the most frequent cause is AIDS that exceeds all aforementioned causes. As age increases, neoplasms and circulatory system diseases become more statistically significant [4]. In our study (Table 2) deaths were caused first by circulatory system diseases (33.80%), followed by traumas and poisoning (21.13%), and by respiratory system diseases (n = 16, 11.27%).

Circulatory system diseases are also the most frequent cause of death in the general population. Various sources report that 80–90% of the homeless are smokers, irrespective of whether the study was conducted in Europe, America, or Asia [46–48]. Smoking is evidently a risk factor for circulatory and respiratory systems diseases. According to the data collected in the same region as our study, 84.96% of the homeless are smokers with the average risk of 27.79 pack-years [8]. This seems to be confirmed by the data included in the first part of Table 2, presenting smoking-attributable deaths. Every second death recorded in our study may be associated with smoking. It should also be noted that the average age at death attributable to smoking is relatively advanced (exposure time). Similar to the study by Hwang SW et al., pleonasms and circulatory system diseases as the causes of death are more frequent in the elderly homeless [4]. A small number of deaths due to infectious diseases (n = 4), noted in the results section, can be attributed to local specificity. In this region, HIV infections in this subpopulation are not particularly frequent (2.36% of infected individuals), whereas a rather high (2–3%) incidence of tuberculosis is reflected in a relatively large number of deaths (n = 3; 2.11%) [49–51].

In their meta-analysis, Fazel et al. determined the percentages of individuals dependent on alcohol at 8.5–58.1%., pointing out one important fact that the percentages of alcohol-dependent individuals among the homeless are generally higher in Europe than in the United States [12]. Romaszko et al. estimated that 78.57% of Polish homeless were dependent on alcohol [8]. In our study, alcohol related deaths amount up to 19.72%. However, if we assume that deaths due to hypothermia among the homeless are very likely to be secondary to alcohol consumption, then this value will increase to 23.24%. In our view, irrespective of different qualifications of such deaths by various authors, in this subpopulation such estimation is justified.

Cold related mortality has decreased in most European countries since 1950 [52]. Nevertheless, people who spend much time outdoors (e.g., the homeless) are obviously affected by low temperatures. When planning this study we had assumed that the number of deaths due to hypothermia would be much larger. In the collected data we revealed only 5 cases of death due to hypothermia. This, however, accounts for as much as 3.52% of deaths, whereas according to the Central Statistical Office in the same period and the same locality 13 deaths due to hypothermia were recorded for the general population (0.15% of the total number of deaths). In other words, deaths caused by hypothermia occurred twenty three-fold more often among the homeless (p<0.001). When comparing causes of death among the homeless included in the Housing First program in Philadelphia (USA) and not participating in it, Henwood et al. observe that deaths due to hypothermia (5.6% of causes of death) occur only in the group not included in the program [39]. It should be noted that the climate in Philadelphia is milder than that in the locality analyzed in our study. In climate conditions of north-east Poland there are no thermally neutral days in wintertime, and cold stress of various intensity occurs almost every day, which makes the homeless permanently exposed to cold stress. Consequently, the probability of death for a homeless person under cold stress is much higher (RR = 3.21) as compared to thermoneutral conditions (Table 4). Moderate cold stress is responsible for a considerable risk for mortality among the homeless (RR = 1.81), while heat stress poses no such risk. A relative death risk under heat stress ac compared to heat comfort is statistically significant, yet definitely lower (RR = 0.37).

The performed TWCA (Fig 3) indicates that seasonal correlations with causes of death combined into groups A and B (Table 2) are noticeable and warrant a reliable interpretation of the examined phenomenon. An evident increase in the number of deaths due to external causes (Fig 3A and 3C) is conditioned by alcohol consumption, which–in combination with the number of deaths due to hypothermia–creates clusters corresponding to winter months. This distribution is more evident when deaths potentially related to smoking are excluded from the analysis (Fig 3A), a factor that most strongly masks the analysis results (Fig 3B). Nevertheless, even then the cluster of causes of deaths termed in this study as “alcohol related death + cold” is most strongly manifested in December (Fig 3B). We believe that this is the result of the combination of meteorological factors (increased number of frosty days, strong winds) and cultural factors (Christmas and elevated alcohol consumption). In group B, circulatory system diseases, which are the most frequent cause of death among the homeless according to our data (Table 2), are quite equally distributed over the year, except for May, as is presented in Fig 3C. Our findings are then consistent with those published by Marti-Soeler et al., who revealed seasonal fluctuations in the number of deaths due to cardiovascular causes in the general population, increasing in winter and decreasing in warmer summer months [53]. GLM analysis (Table 5) confirms that both tmax and tmin are significant factors correlated with some groups of death causes among the homeless, like smoking attributable deaths. Nevertheless, they are not significantly related to cardiovascular deaths. A relation between UTCI and cold related deaths is strongly evident here. Our study examined the association between thermal factors and deaths among the homeless based on the exposure–response relationships with the use of the GLM approach. This study investigated the interactive effects of various meteorological factors on deaths, which improves our understanding of the association between weather conditions and mortality among the homeless caused by different diseases. More importantly, findings from this study may have some important public health implications.

### Conclusions

Our findings point to excessive death rates among the homeless. The average life span of a homeless person is shorter by about 17.5 years from that in the general population. This difference is even more evident among women and amounts to 25.3 years. Although there is no statistically significant difference between the average age at death of a homeless woman and a homeless man, it appears that the tendency to equalize (if not inversion) as compared to the general population is observed here. As in the general population, the most frequent causes of death are related to circulatory system diseases. A large percentage of deaths attributable to smoking and alcohol consumption should be noted. The analysis of relationships between mortality among the homeless and meteorological factors indicates that in conditions characteristic for the temperate transitional climate of Central Europe, such parameters as tmax, tmin and UTCI have both an direct (cold) and indirect (smoking, alcohol) impact on mortality among the homeless. Deaths due to hypothermia were thirteen-fold more frequent among the homeless as compared to the general population. A relative risk of death for a homeless person exposed to cold stress (even moderate one) is much higher as compared to thermoneutral conditions. Our results showed that UTCI may serve as a useful tool to predict death risk in this group of people. It also indicates that weather-warning systems should be implemented to alert the public not only to the danger of extreme weather conditions but should be extended to moderate cold stress attributes and focused on susceptible subpopulations.

### Limitations

In our study a homeless person is defined as any person who ever remained overnight at the Shelter for the Homeless in Olsztyn during the years 2010–2016. Such a person at that time fulfilled the ETHOS classification criteria (category 3.1)[54]. It should be remembered, however, that some homeless people may have never appeared in the shelter (roofless homeless), may have lived and died anywhere within the city of Olsztyn. We are unable to identify such people. Another point that should be taken into consideration when analyzing our data is the fact that homelessness is not a constant parameter. A person who was homeless in 2010, may have died a few years later as a patient in a nursing home, and yet is recorded in our database. Moreover, although homeless people constitute the poorest social class, it is possible that in singular cases particular individuals may have changed their economic status in the analyzed period. We believe, however, that if such an error actually exists, it is minimal.

## Supporting information

S1 Data Aggregate dataset. (XLSX)
